# Therapeutic Potential of Allomyrinasin in Oral Squamous Cell Carcinoma via Decreased NBC Activity

**DOI:** 10.3390/pharmaceutics18050622

**Published:** 2026-05-19

**Authors:** Septika Prismasari, Hyeong Jae Kim, Jeong Hee Hong, Jung Yun Kang

**Affiliations:** 1 Department of Dental Hygiene, College of Health Sciences, Yonsei University, Wonju 26493, Republic of Korea; septika.p@yonsei.ac.kr; 2 Department of Preventive and Community Dentistry, Universitas Gadjah Mada, Yogyakarta 55281, Indonesia; 3 Department of Physiology, College of Medicine, Gachon University, Incheon 21999, Republic of Korea

**Keywords:** allomyrinasin, anticancer, antimicrobial peptide, cell migration, intracellular pH, oral squamous cell carcinoma, reactive oxidative species, sodium bicarbonate cotransporter

## Abstract

**Background/Objectives**: Allomyrinasin is a cationic antimicrobial peptide derived from *Allomyrina dichotoma* larvae with known antibacterial and anti-inflammatory properties; however, its effects on migration-related mechanisms in oral squamous cell carcinoma (OSCC) remain poorly understood. This study investigated the anti-migratory potential of allomyrinasin in OSCC cells, focusing on Na^+^/HCO_3_^−^ cotransporter (NBC) activity as a key migratory module. **Methods**: NBC activity was assessed in YD-38 OSCC cells treated with allomyrinasin. Cell migration was evaluated by wound healing and Transwell assays, and MMP expression. Intracellular reactive oxygen species (ROS), apoptosis-related markers, and lamin A/C expression were analyzed using fluorescence-based assays and gene expression analysis. **Results**: Allomyrinasin inhibited NBC activity and suppressed cell migration without substantial loss of cell viability. *MMP-13* was selectively downregulated among the tested MMPs. Lamin A/C expression was markedly upregulated, suggesting enhanced nuclear stiffness that may restrict confined cell migration. Intracellular ROS levels were elevated, and apoptotic progression was confirmed by increased Annexin V/PI positivity along with downregulation of B-cell lymphoma 2 (*BCL2*) and upregulation of BCL-2–associated X genes (*BAX*), through a p53-independent pathway consistent with the *TP53*-deleted status of YD-38 cells. **Conclusions**: Allomyrinasin suppresses OSCC cell migration by targeting NBC activity as a key component of the migratory machinery, accompanied by oxidative stress induction and pro-apoptotic signaling. These findings identify allomyrinasin as a potential anti-migratory therapeutic candidate and highlight NBC activity as a promising target for attenuating cancer metastasis.

## 1. Introduction

Cationic antimicrobial peptides (AMPs) have attracted attention as anticancer agents because they preferentially interact with the negatively charged surfaces of cancer cells, which are enriched with molecules such as phosphatidylserine, O-glycosylated mucins, and heparan sulfate proteoglycans [1,2]. Among these peptides, allomyrinasin is a small cationic AMP identified from *Allomyrina dichotoma* larvae with potent antibacterial, anti-biofilm, and anti-inflammatory activities, including membrane permeabilization and suppression of inducible nitric oxide synthase (iNOS), cyclooxygenase-2 (COX-2), and pro-inflammatory cytokines in stimulated macrophages [3]. These findings suggest that allomyrinasin is biologically active and membrane-responsive; however, its potential effects on oral cancer have not yet been investigated. The absence of such data represents a missing link, as other AMPs, such as NRC-03 and piscidin-1, have been shown to exert anticancer activity in oral squamous cell carcinoma (OSCC) by inducing mitochondrial oxidative stress and sustained reactive oxygen species (ROS) accumulation [4,5]. However, no study has examined whether allomyrinasin affects the migration-related biology that supports OSCC progression, in addition to its potential effects on cancer cell survival.

OSCC accounts for the majority of oral cancers and remains clinically significant because patient survival remains poor, particularly when the disease is diagnosed after local invasion has already occurred [6]. Cancer cell migration is not governed by a single pathway, but by the coordinated regulation of apoptosis resistance, extracellular matrix (ECM) degradation, nuclear deformability, and intracellular ion homeostasis [7]. These processes are shaped by microenvironmental conditions, including altered pH dynamics and oxidative stress, both of which are known to influence cancer cell survival, motility, and invasive behavior [8,9]. Cancer cells maintain an abnormal pH gradient that promotes proliferation and migration and supports adaptation to a hostile microenvironment [8,10]. Accordingly, targeting ion transporters that regulate intracellular pH has recently emerged as a potential pharmacological strategy in cancer therapy [11]. Among these transporters, the Na^+^/HCO_3_^−^ cotransporter (NBC) plays an important role in maintaining intracellular pH homeostasis and regulating cancer cell motility [12,13].

Similarly, redox imbalance can modulate apoptotic signaling pathways [14], and regulate the expression of migration-related molecules, including MMPs, which contribute to ECM remodeling [9,15]. In addition, changes in nuclear structure and mechanics are increasingly recognized as important determinants of the ability of cancer cells to move through confined tissue spaces during invasion [16,17]. Collectively, these observations suggest that effective anti-migratory strategies may require interference with multiple cellular systems rather than simple induction of cell death alone.

Despite the growing interest in antimicrobial peptides as anticancer agents, most studies have focused primarily on their cytotoxic and pro-apoptotic effects [1,2,18], whereas their influence on migration-related mechanisms has not been thoroughly investigated [19]. Despite its reported antibacterial, anti-biofilm, and anti-inflammatory activities [3], the relevance of allomyrinasin in oral cancer biology remains unknown. Therefore, the present study focused on allomyrinasin as a candidate anticancer peptide for OSCC cells and evaluated its effects on cell migration and molecular events associated with intracellular pH, apoptotic signaling, ECM remodeling, and nuclear structural regulation. From this perspective, allomyrinasin exhibits broader biological activity than membrane disruption alone and provides useful insights into migration-related mechanisms in OSCC.

## 2. Materials and Methods

### 2.1. Peptide

Allomyrinasin was synthesized by solid-phase peptide synthesis and obtained from Anygen Co., Ltd. (Gwangju, Republic of Korea). The lyophilized peptide was reconstituted in distilled water and stored at −20 °C until further use.

### 2.2. Cell Culture

The human OSCC cell line YD-38 (lower gingiva origin, *TP53*-deleted/p53-null) was obtained from the Korean Cell Line Bank [20,21,22]. Cells were maintained in the RPMI 1640 medium (HyClone, Logan, UT, USA) supplemented with 10% fetal bovine serum (FBS; Gibco, USA) and 1% penicillin–streptomycin (Gibco, Grand Island, NY, USA) at 37 °C in a humidified atmosphere of 5% CO_2_. Cells were passaged using 0.25% trypsin-ethylenediaminetetraacetic acid (Trypsin-EDTA; Bandio Bioscience, Pocheon, Republic of Korea).

### 2.3. NBC Activity Measurement

Sodium bicarbonate co-transporter (NBC) activity was quantified by measuring pHi recovery kinetics using the ratiometric pH-sensitive fluorescent dye 2′,7′-bis-(2-carboxyethyl)-5(and-6)-carboxyfluorescein acetoxymethyl ester (BCECF-AM; Invitrogen, Carlsbad, CA, USA) [23]. Following allomyrinasin treatment, cells were transferred to a temperature-controlled perfusion chamber mounted on an inverted fluorescence microscope and maintained at 37 °C throughout imaging. Cells were loaded with 20 µM BCECF-AM in the presence of 0.1% Pluronic F-127 for 15 min at room temperature under dark conditions, after which they were washed and perfused with physiological salt solution to stabilize fluorescence signals before measurement. BCECF fluorescence was captured using alternating excitation wavelengths of 440 nm and 495 nm, with emission collected at 530 nm; pHi was continuously monitored as the F495/F440 fluorescence ratio. Images were acquired at 1 s intervals using a charge-coupled device camera and subsequently analyzed using the MetaFluor software version 7.0 (Molecular Devices, San Jose, CA, USA). Background fluorescence was subtracted from raw signals at each respective wavelength before ratio computation.

To evaluate HCO_3_^−^-dependent, Na^+^-driven pHi recovery attributable to NBC activity, cells were sequentially perfused with (i) a standard physiological solution, (ii) a HCO_3_^−^/CO_2_-buffered solution, and (iii) a Na^+^-free HCO_3_^−^/CO_2_-buffered solution. Na^+^/H^+^ exchanger activity was pharmacologically suppressed by incorporating 10 µM 5-(N-ethyl-N-isopropyl) amiloride (EIPA; Sigma-Aldrich, St. Louis, MO, USA) into all measurement solutions. Intracellular acidification was induced by transient exposure to a CO_2_-saturated HCO_3_^−^ solution, after which cells were maintained in Na^+^-free HCO_3_^−^ solution to preclude Na^+^-dependent pHi recovery before initiation of the NBC measurement phase. NBC-mediated intracellular alkalinization was subsequently initiated by switching to a Na^+^-containing HCO_3_^−^/CO_2_-buffered solution; NBC activity was defined as the initial rate of pHi increase (ΔpHi/Δt), calculated from the slope of the fluorescence ratio during the first 30–45 s following Na^+^ reintroduction.

### 2.4. Wound Healing Scratch Assay

Confluent YD-38 monolayers grown in a six-well plate were scratched using a 200 µL pipette tip. The medium was replaced with low-serum RPMI medium (1% FBS) containing allomyrinasin concentration. Images were captured at 0 and 24 h. The wound closure percentage was calculated after measuring the gap using ImageJ software version 1.54 (National Institutes of Health, Bethesda, MD, USA).

### 2.5. Transwell Migration Assay

Cell invasion was evaluated using Transwell polycarbonate inserts (6.5 mm diameter, 8.0 μm pore size, 24-well format). YD-38 cells were seeded into the upper chamber at a density of 5 × 10^4^ cells/insert in 200 μL RPMI medium containing low serum (1% FBS). The lower chamber was filled with 500 μL RPMI supplemented with 10% FBS as a chemoattractant. Allomyrinasin (50 μg/mL) was applied during the migration period, and the cells were incubated for 4 h at 37 °C in 5% CO_2_. Cells that migrated to the lower surface were fixed with pre-chilled methanol at −20 °C for 1 min, washed with Dulbecco’s phosphate-buffered saline (DPBS), and stained with 4′,6-diamidino-2-phenylindole (DAPI) in the dark. The membranes were washed and observed under an LSM 700 Zeiss confocal microscope (Fluoview; CarlZeiss, Jena, Germany). Migration was quantified by counting DAPI-positive nuclei in at least five random fields per insert, and the results were expressed relative to the control group. This protocol is adapted from Ji et al. [24].

### 2.6. Reverse Transcription Quantitative Polymerase Chain Reaction (RT-qPCR)

Total RNA was isolated from OSCC cells (YD-38) after treatment with allomyrinasin peptide (e.g., 50 and 100 µg/mL) for 24 h. Cells were lysed directly in culture dishes using a phenol/guanidinium thiocyanate reagent (RiboEx; GeneAll, Seoul, Korea) according to the manufacturer’s instructions. Briefly, chloroform was added to the lysate, samples were vigorously mixed and centrifuged at 12,000× *g* for 15 min at 4 °C to separate phases, and the aqueous phase was collected. The RNA was resuspended in RNase-free water. RNA concentration and purity were assessed using a NanoDrop spectrophotometer (ND-1000; Thermo Fisher Scientific, Waltham, MA, USA), and samples meeting purity criteria (A260/280 within the acceptable range) were used for downstream analysis.

Complementary DNA (cDNA) was synthesized from equal amounts of total RNA using a reverse transcription kit (RocketScript™ RT PreMix; Bioneer, Daejeon, Korea). Quantitative PCR was performed using SYBR Green chemistry (PowerUp™ SYBR™ Green Master Mix; Applied Biosystems, Waltham, MA, USA) on a real-time PCR system (QuantStudio™ 3; Applied Biosystems). The cycling conditions were uracil-DNA glycosylase (UDG) activation at 50 °C for 2 min, polymerase activation/initial denaturation at 95 °C for 2 min, followed by 40 cycles of denaturation at 95 °C for 15 s, annealing at 55–60 °C for 15 s (primer-dependent), and extension at 72 °C for 1 min. Melt-curve analysis was performed at the end of amplification to verify single-product specificity. Target gene expression (*TP53*, *BAX*, *BCL2*, MMP-related genes) was normalized to the housekeeping gene *GAPDH* and calculated using the 2^(−ΔΔCt)^ method. Primer sequences are provided in Table 1, and all reactions were run in technical triplicate with at least three independent biological replicates.

### 2.7. Cell Viability Assay

Cell viability was assessed using the 3-(4,5-dimethylthiazol-2-yl)-5-(3-carboxymethoxyphenyl)-2-(4-sulfophenyl)-2H-tetrazolium (MTS) assay (G3580, Promega, Madison, WI, USA). YD-38 cells were seeded in 96-well plates, incubated overnight, and treated with allomyrinasin at 50, 100, and 200 µg/mL for 24 h at 37 °C. The MTS reagent was added and incubated for 1 h, and the absorbance at 490 nm was recorded using a SpectraMax ABS microplate reader.

### 2.8. Reactive Oxygen Species (ROS) Assay

Cells at 60–70% confluency were treated with allomyrinasin (50 and 100 µg/mL) for 30 min. PBS and H_2_O_2_ served as negative and positive controls, respectively. Cells were loaded with 2′,7′-dichlorofluorescin diacetate (DCFH-DA; Invitrogen, USA) fluorescent probe for 30 min. Fluorescence (excitation/emission at 430/530 nm) was captured using an Olympus CKX53 fluorescence microscope (Olympus, Tokyo, Japan) at a fixed exposure time of 50 ms and quantified using the ImageJ software version 1.54 (National Institutes of Health, Bethesda, USA). Fluorescence images were converted to an 8-bit format using ImageJ software (National Institutes of Health, Bethesda, USA), and the intensity thresholds were adjusted with reference to the original images. The area reflecting fluorescence above the threshold was measured for each field. Values were normalized to the control group by dividing the measured fluorescence intensity of each treatment condition by the mean fluorescence intensity of untreated control cells. The detector settings were maintained constant across all conditions to enable direct comparison.

### 2.9. Annexin V-Fluorescein Isothiocyanate (FITC)/Propidium Iodide (PI) Apoptosis Assay

YD-38 cells (30,000/well, 96-well plates) were treated with allomyrinasin (50 and 100 µg/mL) for 6 h and stained using the Annexin V-FITC Apoptosis Detection Kit (eBioscience™, San Diego, CA, USA) modified protocol for microscopy imaging [25]. FITC and PI fluorescence were captured using an Olympus CKX53 microscope (Olympus, Tokyo, Japan) with a 40× water immersion objective. Following Sriboonaied et al. [26], fluorescence intensity was quantified using the corrected total cell fluorescence (CTCF), calculated as:


CTCF = integrated density − (area of selected cell × mean fluorescence of background).

### 2.10. Immunofluorescence Staining of Lamin A/C

Immunofluorescence staining was performed to assess treatment-induced alterations in nuclear envelope integrity, as reflected by changes in lamin A/C expression, in YD-38 cells. Cells were seeded onto sterile glass coverslips placed within a 35 mm culture dish and cultured until reaching approximately 60–80% confluency, after which they were treated with allomyrinasin peptide (50 µg/mL) or the respective control for 24 h.

Following treatment, cells were rinsed with ice-cold DPBS and fixed with pre-chilled methanol (−20 °C) for 10 min. The coverslips were subsequently washed three times with DPBS to remove residual fixative. Non-specific antibody binding was blocked by incubating the coverslips in a blocking buffer consisting of 0.5% bovine serum albumin (Bovogen Biologicals, Kellor East, VIC, Australia) in DPBS, optionally supplemented with 10% normal goat serum, for 1 h at room temperature. Next, the cells were incubated overnight at 4 °C with a primary antibody directed against lamin A/C (ab185014, Abcam, Cambridge, UK), diluted in the blocking buffer. The following day, the coverslips were washed three times with DPBS. Coverslips were mounted onto glass slides using an antifade mounting medium (Fluoromount-G, with or without DAPI depending on the experimental workflow) and allowed to fully cure before imaging.

Fluorescence images were acquired using a laser scanning confocal microscope (Zeiss LSM 700, Jena, Germany) under standardized acquisition parameters and were maintained consistently across all experimental groups within each experiment. A minimum of three randomly selected fields per coverslip were imaged for each condition, and all experiments were performed independently using separate biological replicates. Lamin A/C fluorescence intensity was quantitatively analyzed using the Metamorph software version 7.0 (Molecular Devices, San Jose, CA, USA) by measuring the mean fluorescence intensity and integrated density for each defined region.

### 2.11. Statistical Analysis

Statistical analyses were performed using GraphPad Prism 8.0 (GraphPad Software, San Diego, CA, USA). Multiple-group comparisons were performed using one-way analysis of variance (ANOVA) with post hoc tests, and two-group comparisons were performed using unpaired Student’s *t*-tests. Data are presented as mean ± SD. A *p*-value < 0.05 was considered significant.

## 3. Results

### 3.1. Allomyrinasin Reduces NBC Activity in OSCC Cells

**Figure 1:**
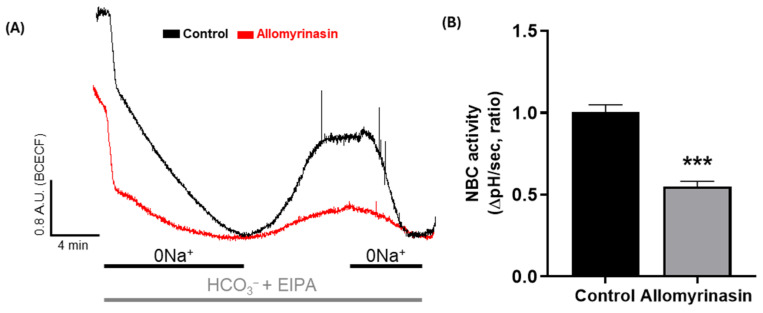
Allomyrinasin suppresses NBC activity in YD-38 OSCC cells. (**A**) NBC activity in the YD-38 cells with 50 μg/mL allomyrinasin (red line) and control (black line) at 24 h. Average traces are presented. (**B**) The bars indicate the mean ± SEM of data. *** *p* < 0.001 versus control.

Given that the NBC plays a critical role in maintaining intracellular pH homeostasis and regulating cancer cell motility [12], NBC activity was evaluated as a migratory module in YD-38 cells derived from gingival squamous cell carcinoma [27]. Allomyrinasin was applied at 50 μg/mL, the lowest concentration at which biological activity was demonstrated in a prior study characterizing the anti-inflammatory properties of this peptide [3], for 24 h to allow sufficient time for the peptide to interact with membrane-associated transport machinery and to evaluate the sustained inhibitory effect on transporter activity. Representative traces showed that cells treated with allomyrinasin exhibited a weaker recovery pattern than the untreated control cells (Figure 1A). Quantitative analysis confirmed that NBC activity was reduced by allomyrinasin treatment (Figure 1B). These findings indicate that allomyrinasin reduced NBC activity in OSCC cells.

### 3.2. Allomyrinasin Suppresses Migratory Behavior of OSCC Cells

To assess whether allomyrinasin affects the migration of OSCC cells, wound healing and transwell migration assays were performed using YD-38 cells. Wound healing assays were performed at 50 and 100 μg/mL over 24 h, a standard duration for evaluating monolayer closure in scratch assays. Transwell migration was assessed at 50 μg/mL for 4 h, a timeframe selected to capture active chemotactic migration while minimizing potential confounding effects from cell proliferation.

**Figure 2:**
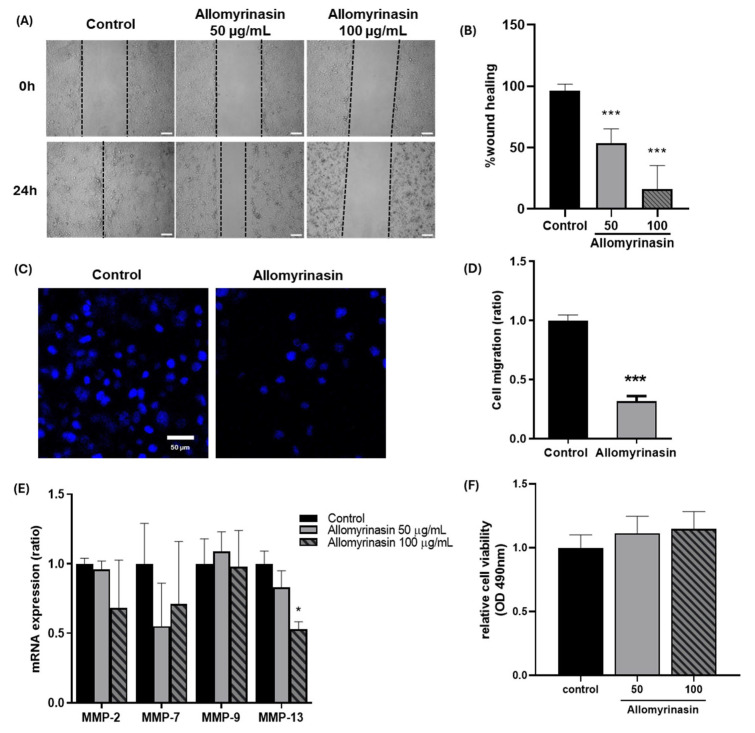
Allomyrinasin inhibits migratory behavior and reduces *MMP-13* expression without significantly affecting cell viability in YD-38 OSCC cells. (**A**) Representative wound healing images of YD-38 cells at 0 h and 24 h under control conditions or after treatment with allomyrinasin at 50 or 100 μg/mL. Scale bar: 30 µm. (**B**) Quantification of wound healing percentage. (**C**) Representative images from the cell migration assay in the control group and the allomyrinasin-treated group after 4 h. Scale bar: 50 µm. (**D**) Quantification of cell migration ratio. (**E**) Relative mRNA expression of *MMP-2*, *MMP-7*, *MMP-9*, and *MMP-13* in cells after treatment with allomyrinasin for 24 h. (**F**) Relative cell viability measured by OD490 after treatment with 50 and 100 μg/mL allomyrinasin for 24 h. Data are presented as mean ± SEM. * *p* < 0.05, *** *p* < 0.001 versus control.

In the wound healing assay, the control group demonstrated marked closure of the scratched area at 24 h, whereas cells treated with allomyrinasin at 50 and 100 μg/mL exhibited delayed wound closure (Figure 2A). Quantitative analysis confirmed that wound closure was significantly reduced in a dose-dependent manner, with decreases of approximately 50% at 50 μg/mL and 85% at 100 μg/mL relative to the control group (Figure 2B). Consistent with these findings, Transwell migration assays revealed a marked reduction in the number of migrated cells following allomyrinasin treatment (Figure 2C,D). These results indicate that allomyrinasin suppressed the migratory capacity of OSCC cells.

To determine whether the observed reduction in migration was accompanied by changes in ECM remodeling, the expression of migration-related MMPs was examined. Allomyrinasin treatment showed a decreasing trend in *MMP-2* and *MMP-7* expressions, whereas *MMP-9* expression remained unchanged (Figure 2E). In contrast, *MMP-13* expression was significantly reduced following allomyrinasin treatment, as shown in Figure 2E, suggesting that the anti-migratory effect of allomyrinasin may be associated more closely with *MMP-13* downregulation than with broad suppression of all tested MMPs.

To exclude the possibility that reduced migration simply reflects cytotoxicity, relative cell viability was assessed after longer exposure to allomyrinasin. No marked reduction in cell viability was observed under the tested conditions (Figure 2F), indicating that the inhibitory effect on cell migration was not primarily attributable to loss of cell viability.

### 3.3. Allomyrinasin Increases Lamin A/C Expression in OSCC Cells

Nuclear structural properties are important determinants of cancer cell deformability and migration [16,17]. To investigate whether allomyrinasin was associated with changes in nuclear structural components, lamin A/C expression was examined by immunofluorescence staining. Assessment was performed after treatment with 50 μg/mL allomyrinasin for 24 h, as this concentration and duration allowed sufficient time for protein-level changes to manifest while ensuring that early apoptosis rather than late apoptosis predominated, thereby preserving an adequate population of adherent cells for immunofluorescence analysis.

**Figure 3:**
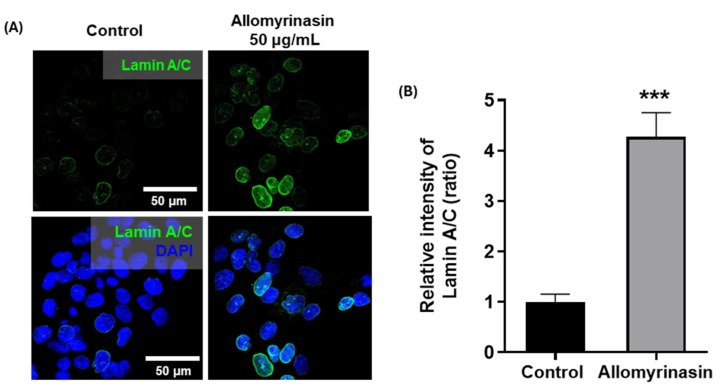
Allomyrinasin increases Lamin A/C expression in YD-38 OSCC cells. (**A**) Representative immunofluorescence images of Lamin A/C in control and 50 μg/mL allomyrinasin-treated OSCC cells for 24 h. Nuclei were counterstained with DAPI. Scale bar: 50 µm. (**B**) Quantification of relative Lamin A/C fluorescence intensity. Data are presented as mean ± SEM. *** *p* < 0.001 versus control.

Compared to the control group, allomyrinasin-treated cells demonstrated approximately 4.2-fold stronger lamin A/C fluorescence (Figure 3A). Quantitative analysis revealed a significant increase in the relative intensity of lamin A/C after treatment (Figure 3B). These results indicate that allomyrinasin was associated with increased lamin A/C expression in YD-38 cells, suggesting a potential increase in nuclear stiffness.

### 3.4. Allomyrinasin Increases Intracellular ROS Accumulation in OSCC Cells

Oxidative stress is known to regulate cancer cell behavior through stress-responsive signaling pathways [9,14]. To determine whether allomyrinasin induces an oxidative response in OSCC cells, intracellular ROS levels were assessed after short-term exposure using DCFH-DA staining. ROS levels were measured after treatment with allomyrinasin at 50 and 100 μg/mL for 30 min. The exposure time of 30 min was selected to capture the early oxidative response as a proximal event, before downstream signaling cascades and secondary cellular responses could confound the measurement of direct ROS induction.

**Figure 4:**
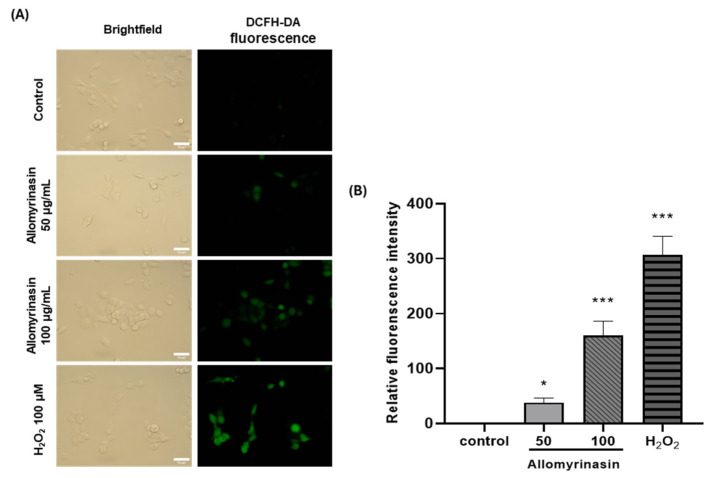
Allomyrinasin increases intracellular ROS accumulation in YD-38 OSCC cells. (**A**) Representative brightfield and DCFH-DA fluorescence images of cells under control conditions, after treatment for 30 min with allomyrinasin at 50 or 100 μg/mL or with H_2_O_2_ as a positive control. Scale bar: 10 µm. (**B**) Quantification of relative DCFH-DA fluorescence intensity. Values represent the fluorescence-positive area of treated cells normalized to the mean value of fluorescence of untreated control cells (set as 1.0). Data are presented as mean ± SD. * *p* < 0.05, *** *p* < 0.001 versus control.

Control cells exhibited weak basal fluorescence consistent with low resting ROS levels, whereas allomyrinasin-treated cells displayed visibly increased fluorescence intensity in a concentration-dependent manner, and H_2_O_2_-treated cells, as a positive control, showed the strongest signal (Figure 4A). Quantitative analysis revealed that allomyrinasin caused a statistically significant and concentration-dependent increase in DCFH-DA fluorescence intensity. At 50 μg/mL, ROS levels were significantly elevated compared to those in control cells (approximately 40.6-fold), and treatment at 100 μg/mL induced a further significant increase (approximately 175.4-fold), with H_2_O_2_ producing the highest response (Figure 4B). These findings indicate that allomyrinasin increases intracellular ROS levels and supports the presence of a ROS-associated cellular response in OSCC cells. The large fold-change values partially reflect the very low basal DCFH-DA fluorescence in untreated YD-38 cells; absolute fluorescence intensities remained below the detector saturation thresholds across all conditions.

### 3.5. Allomyrinasin Induces Apoptosis in OSCC Cells

Disruption of NBC activity and oxidative stress are known to increase the susceptibility of cancer cells to apoptosis [9,13,28]. To examine whether these responses were accompanied by apoptotic changes, Annexin V/PI staining and apoptosis-related gene expression were analyzed in OSCC cells. Annexin V/PI staining was performed after treatment with allomyrinasin at 50 and 100 μg/mL for 6 h, a timepoint selected to capture intermediate-stage apoptotic progression based on the kinetics of phosphatidylserine externalization. Gene expression of apoptosis-related markers was assessed at 24 h to allow sufficient time for the transcriptional responses to manifest.

**Figure 5:**
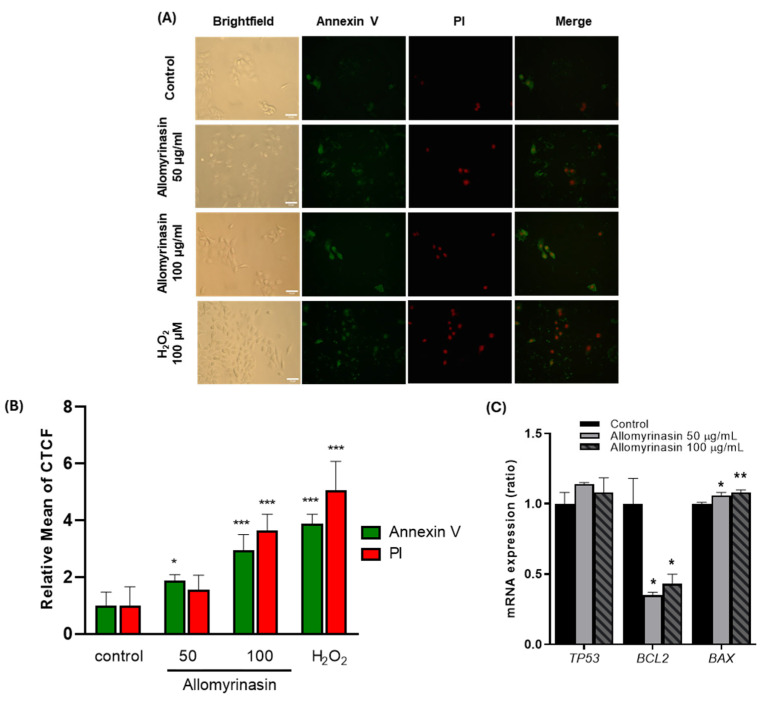
Allomyrinasin induces sequential apoptotic progression and modulates apoptosis-associated gene expression in YD-38 OSCC cells. (**A**) Representative brightfield, Annexin V-FITC (green), propidium iodide (PI; red), and merged fluorescence images of cells under control conditions, after treatment with allomyrinasin at 50 or 100 μg/mL for 6 h, or with H_2_O_2_ as a positive control. Scale bar: 10 µm. (**B**) Quantitative analysis of Annexin V and PI fluorescence expressed as relative mean corrected total cell fluorescence (CTCF), normalized to control. Values for each condition were divided by the mean CTCF of the control group to generate relative values. (**C**) Relative mRNA expression of *TP53*, *BCL2*, and *BAX* after treatment with allomyrinasin for 24 h, normalized to *GAPDH*. Data are presented as mean ± SEM from *n* ≥ 3 independent experiments. * *p* < 0.05, ** *p* < 0.01, *** *p* < 0.001 versus control.

Annexin V/PI fluorescence microscopy revealed concentration-dependent increases in apoptotic markers after treatment with allomyrinasin for 6 h (Figure 5A). Quantitative analysis using CTCF demonstrated distinct patterns of apoptotic marker expression at different concentrations (Figure 5B). At 50 μg/mL, annexin V fluorescence was elevated (approximately 1.9-fold increase; *p* < 0.05), whereas PI fluorescence showed only a modest, non-significant increase (approximately 1.7-fold). Phosphatidylserine externalization without a corresponding loss of membrane integrity is a characteristic of early apoptosis. At 100 μg/mL, both markers were elevated compared with control cells, with annexin V showing an approximately 2.9-fold increase (*p* < 0.001) and PI demonstrating an approximately 3.9-fold increase (*p* < 0.001). The higher PI signal relative to the annexin V signal at this concentration indicates progression to late apoptotic stages. H_2_O_2_-treated positive control cells exhibited robust increases in both markers (approximately 3.9-fold and 4.5-fold for annexin V and PI, respectively; *p* < 0.001).

To further characterize this response at the transcriptional level, the expression of key apoptosis-regulatory genes was examined by RT-qPCR after treatment with allomyrinasin for 24 h. The expression of the tumor suppressor p53 remained unchanged across treatment groups, suggesting the involvement of p53-independent apoptotic mechanisms (Figure 5C). In contrast, *BCL2* expression was reduced following allomyrinasin treatment at both concentrations (*p* < 0.05), whereas *BAX* expression was correspondingly increased (*p* < 0.05 at 50 μg/mL; *p* < 0.01 at 100 μg/mL). Collectively, these findings indicate that allomyrinasin induces dose-dependent apoptotic progression in OSCC cells, characterized by early apoptotic changes at lower concentrations, which advance to late apoptosis at higher concentrations. The sequential pattern of early-to-late apoptotic progression, combined with p53-independent modulation of Bcl-2 family proteins, supports the engagement of the canonical intrinsic apoptotic pathway.

## 4. Discussion

The findings of the present study demonstrate that allomyrinasin exerts anti-cancer effects in OSCC cells by suppressing migration through the inhibition of NBC activity as a key migratory machinery, accompanied by oxidative stress induction and activation of pro-apoptotic signaling. The approximately 45% reduction in NBC activity represents a potential mechanism for this insect-derived peptide, consistent with emerging evidence that bicarbonate transporters are critical therapeutic targets in cancer [10,13,28]. Cancer cells maintain an alkaline intracellular pH despite acidic microenvironments through upregulated acid-extruding mechanisms, including NBC transporters, and disrupting this reversed pH gradient compromised cell proliferation, migration, and survival [8,12]. Our findings extend previous observations in breast cancer [28,29] to oral cancer, demonstrating that naturally derived peptides achieve functional NBC inhibition. Among the NBC isoforms, the electroneutral Na^+^,HCO_3_^−^ cotransporter NBCn1 (*SLC4A7*) has been identified as a key contributor to net acid extrusion in cancer, with its protein expression upregulated during breast carcinogenesis and its genetic disruption delaying tumor development and decelerating tumor growth in vivo [29]. A recent study demonstrated that pharmacological blockade of NBCn1 using the high-affinity anti-NBCn1 monoclonal antibody 5H2.1, which targets the third extracellular loop of human NBCn1/SLC4A7, suppressed net acid extrusion in human breast cancer tissue and reduced tumor growth in patient-derived xenograft models [28]. Functional characterization of oral epidermoid carcinoma cells confirmed the expression and activity of multiple NBC isoforms, including NBCn1 (SLC4A7) and NBCe1 (*SLC4A4*), in oral cancer cells [30], supporting the relevance of NBC-mediated pH regulation in this tumor type.

Allomyrinasin exhibited potent dose-dependent inhibition of cell migration, with approximately 50% and 85% reduction in wound closure at 50 and 100 μg/mL, respectively, and 70% reduction in Transwell migration. Importantly, the anti-migratory effect occurred without significant cytotoxicity, distinguishing allomyrinasin from conventional cytotoxic agents. Although *MMP-9* expression remained unchanged, *MMP-2* and *MMP-7* expressions displayed non-significant decreasing trends, whereas *MMP-13* expression was significantly reduced. The differential MMP expression pattern provides mechanistic insights into the observed anti-migratory effects. *MMP-13* overexpression correlates with lymph node metastasis, advanced staging, and poor prognosis in OSCC [15,31], suggesting that its suppression contributes to the anti-metastatic potential of allomyrinasin. However, the significant migration inhibition observed at 50 μg/mL despite non-significant *MMP-13* reduction at this concentration indicates that *MMP-13* downregulation represents only one component of a multi-factorial anti-migratory mechanism. The rapid onset of migration inhibition in the Transwell assay performed for 4 h, which is insufficient for transcriptional changes to substantially alter protein levels, suggests the involvement of post-translational mechanisms. NBC transporters have been implicated as key components of the migratory machinery in cancer cells [13]. Intracellular pH critically regulates cytoskeletal dynamics, as numerous actin-binding proteins and focal adhesion components contain pH-sensitive histidine residues that function as molecular switches [32,33]. The connection between NBC inhibition and *MMP-13* suppression may involve pH-sensitive transcription factors such as NF-κB and AP-1, whose activity is compromised by intracellular acidification [34]. Additionally, lamin A/C upregulation in surviving cells increased nuclear stiffness and mechanically constrained confined migration in dense ECM environments. Together, these converging mechanisms involving pH-dependent cytoskeletal regulation, transcriptional suppression of *MMP-13*, and nuclear mechanical constraints explain the robust anti-migratory effects observed across this concentration range. Although the low-serum conditions (1% FBS) used in the wound healing assay minimized proliferative contributions, the consistency between the wound healing and the Transwell assay performed for 4 h further supports genuine anti-migratory activity rather than an anti-proliferative artifact.

The dose-dependent ROS accumulation (40-fold and 175-fold increases at 50 and 100 μg/mL, respectively) reveals an oxidative stress mechanism complementing pH-mediated effects. The observed ROS elevation aligns with reports that antimicrobial peptides induce cancer cell death via mitochondrial oxidative stress [5,35,36]. The combination of increased ROS production and diminished detoxification capacity creates self-amplifying oxidative stress. Cancer cells operate near their maximum tolerable ROS threshold, rendering them more susceptible than normal cells to oxidative stress-inducing agents [9,37]. The high fold-change values partially reflect the minimal basal fluorescence signal in untreated YD-38 cells and should be interpreted in the context of absolute fluorescence measurements. Validation using additional ROS-specific probes would further strengthen these observations.

The apoptosis data demonstrated a dose-dependent sequential progression consistent with canonical intrinsic pathway engagement. Early apoptosis (annexin V^+^/PI^−^) predominated at 50 μg/mL, advancing to late apoptosis (annexin V^+^/PI^+^) at 100 μg/mL. The sequential pattern of phosphatidylserine externalization preceding membrane permeabilization distinguishes allomyrinasin from purely membrane-lytic antimicrobial peptides that induce simultaneous annexin V/PI positivity through direct membrane disruption [38,39], and instead indicates regulated apoptotic cell death [40]. At the molecular level, allomyrinasin treatment downregulated anti-apoptotic *BCL2* and upregulated pro-apoptotic *BAX* while leaving *TP53* expression unchanged, suggesting that the mitochondrial pathway by allomyrinasin is activated in a p53-independent manner. Mechanistically, ROS-mediated regulation provides a direct link between the rapid induction of oxidative stress by allomyrinasin and subsequent Bcl-2 family modulation. Elevated ROS promotes Bcl-2 phosphorylation and ubiquitin-proteasomal degradation while stabilizing pro-apoptotic Bax through altered phosphorylation patterns [41,42]. The resulting shift in the *BAX/BCL2* ratio favors modulation of mitochondrial outer membrane permeabilization and caspase activation. The apoptotic cascade may also be linked to the NBC inhibition observed in the present study. Sustained intracellular acidification resulting from impaired NBC-mediated pH recovery has been shown to sensitize cancer cells to apoptosis by compromising the activity of pH-dependent survival signaling pathways [8,28]. Axelsen et al. [28] demonstrated that pharmacological blockade of NBCn1-mediated Na^+^,HCO_3_^−^ cotransport in breast cancer cells produced pH-dependent growth inhibition and increased apoptosis, supporting the concept that NBC inhibition can function as an upstream event that lowers the apoptotic threshold. The absence of p53 transcriptional response despite significant ROS accumulation is consistent with the established *TP53* status of the YD-38 cell line, which harbors a *TP53* deletion that results in the loss of p53 protein expression [20,21,22]. The constitutive p53 deficiency in YD-38, rather than a stress-nonresponsive mutant p53, provides a clear molecular basis for the p53-independent nature of the apoptotic signaling observed in this study. The high prevalence of *TP53* mutations in HPV-negative OSCC is reported to be 62–80% [43,44], where mutant p53 exhibits constitutive expression unresponsive to genotoxic stress. The p53-independent apoptotic mechanism has substantial therapeutic implications because *TP53* mutations frequently confer resistance to conventional chemotherapeutic agents and radiation therapy in OSCC [45]. Approximately 50% of all human cancers harbor p53 mutations, and agents capable of engaging in apoptosis through alternative, p53-independent mechanisms therefore retain efficacy against a substantial fraction of treatment-refractory tumors [46,47,48].

The 4.2-fold upregulation of lamin A/C expression represents a notable observation with potential mechanistic implications, although direct biophysical measurements of nuclear deformability were not performed in this study. Lamin A/C is the primary determinant of nuclear stiffness, with expression inversely correlating with nuclear deformability and confined migration capacity [16,17,49]. Highly metastatic cancer cells frequently exhibit reduced lamin A/C, conferring nuclear deformability advantageous for squeezing through tissue barriers, whereas increasing lamin A/C expression has been reported to impair confined migration [16,50]. The nucleus constitutes the rate-limiting factor for cell migration through spaces smaller than nuclear diameter, and increased nuclear stiffness physically constrains metastatic dissemination [49]. Because immunofluorescence at 24 h selectively captures adherent surviving cells, the 4.2-fold lamin A/C upregulation observed at 50 μg/mL likely reflects a stress-protective response in cells that resisted apoptotic commitment rather than a uniform population-wide effect. Lamin A/C expression increases under oxidative stress to stabilize nuclear architecture, protect genome integrity [51,52], and promote DNA base excision repair and mitochondrial function through the NAMPT-NAD+ pathway [53]. Critically, although lamin A/C upregulation may enhance cell survival, it simultaneously imposes a substantial migratory penalty. Allomyrinasin treatment, therefore, creates a bifurcated outcome wherein cells either undergo apoptosis or survive with upregulated Lamin A/C that compromises their migratory potential.

**Figure 6:**
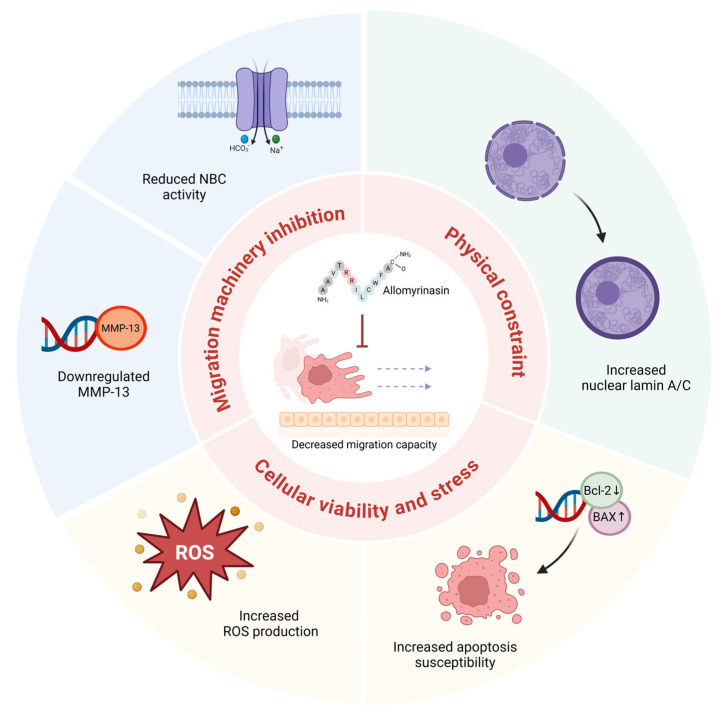
Summary of allomyrinasin-induced cellular responses converging on decreased migration capacity in YD-38 OSCC cells. Allomyrinasin elicits multi-target responses organized into three functional categories. Migration machinery inhibition (upper left) encompasses reduced Na^+^/HCO_3_^−^ cotransporter (NBC) activity, which impairs intracellular pH homeostasis supporting cancer cell motility, and selective *MMP-13* downregulation, which limits extracellular matrix degradation required for tissue invasion. Physical constraint (upper right) involves increased lamin A/C expression consistent with enhanced nuclear stiffness that may restrict cancer cell deformability during confined migration. Cellular viability and stress (lower panel) includes dose-dependent intracellular ROS accumulation and *BAX/BCL2* modulation (*BAX* upregulation, *BCL-2* downregulation), increasing apoptosis susceptibility through a p53-independent mechanism consistent with the *TP53*-deleted status of YD-38 cells. The convergence of these three axes on suppressed migration capacity is depicted at the center, where a cancer cell is shown arrested at the basement membrane barrier.

The present findings can be organized into three functional categories that collectively contribute to the anti-migratory effect of allomyrinasin in OSCC cells (Figure 6). Migration machinery inhibition encompasses NBC-mediated pH dysregulation and *MMP-13* downregulation. Impaired NBC activity disrupts intracellular pH homeostasis, compromising pH-sensitive cytoskeletal dynamics and focal adhesion remodeling essential for directional cell movement [32,33]. Concurrent *MMP-13* suppression limits extracellular matrix degradation required for tissue invasion [15,31]. Physical constraint involves Lamin A/C upregulation and the associated increase in nuclear rigidity. Because the nucleus constitutes the rate-limiting barrier for migration through confined spaces [49], Lamin A/C-mediated increases in nuclear stiffness may physically restrict passage through dense extracellular matrix environments [16,17,50]. Cellular viability and stress encompass ROS accumulation and pro-apoptotic Bcl-2 family modulation. Elevated ROS and the resulting *BAX/BCL2* ratio shift reduce the viable, migration-competent cell population while compromising cellular fitness in surviving cells [9,37,50,54]. The simultaneous engagement of these three functionally distinct categories suggests that allomyrinasin targets multiple cellular systems required for the metastatic phenotype, and the convergence on a common anti-migratory outcome may reduce the likelihood of resistance through single-pathway adaptation [7].

However, several alternative interpretations warrant further investigation. Although the present findings support a model in which NBC inhibition drives ROS accumulation as a proximal signaling event, ROS accumulation may also reflect contributions from additional mechanisms, including mitochondrial dysfunction, suppression of endogenous antioxidant systems, or secondary ROS generation during early apoptotic processes. Similarly, lamin A/C upregulation, while consistent with an anti-migratory response, could additionally reflect a broader cellular stress adaptation. The shift in *BAX/BCL2* ratio observed in this study is consistent with NBC- and ROS-mediated apoptotic signaling. However, the direct effects of allomyrinasin on mitochondrial membrane dynamics should be included as a contributing factor. Furthermore, the anti-migratory effects characterized here are likely multifactorial, and the underlying mechanisms, such as alterations in cell adhesion molecules or cytoskeletal organization, warrant further investigation in future studies.

Several limitations need to be acknowledged in this study. Experiments were conducted in a single cell line (YD-38, lower gingiva origin, *TP53*-deleted), and validation of additional OSCC lines with different genetic backgrounds, including cell lines with wild-type or missense-mutated *TP53*, such as YD-9 and YD-8, is necessary to determine whether the observed responses reflect a broadly shared feature of OSCC biology or are specific to the molecular context of this particular cell line. The use of a single cell line also precludes the assessment of whether allomyrinasin efficacy varies across different anatomical subsites of oral cancer. Mechanistic relationships are correlative; causal verification requires targeted interventions such as antioxidant co-treatment with N-acetylcysteine to assess the requirement of ROS for Bcl-2 family modulation and lamin A/C upregulation, NBC overexpression rescue experiments, or lamin A/C knockdown to establish the downstream signaling hierarchy. The interpretation of population heterogeneity would benefit from single-cell flow cytometric analysis. Several assays in this study were performed at single concentrations, limiting dose–response characterization. Different timepoints were employed across assays to capture distinct phases of the cellular response; although this approach is consistent with the proposed temporal hierarchy of ROS as a proximal trigger, it does not permit direct causal inference within a single timeframe.

The in vitro design does not address pharmacokinetics, biodistribution, or in vivo efficacy, and specific molecular targets of allomyrinasin remain uncharacterized. Changes in apoptosis-related gene expression (*TP53*, *BAX*, *BCL2*) were assessed at the mRNA level by RT-qPCR without corresponding protein-level validation by Western blotting. Although the mRNA expression changes for these well-characterized genes may not fully correspond to the direction of protein-level alterations, post-transcriptional regulation may modify the magnitude or kinetics of protein changes. Furthermore, the interpretation of Lamin A/C upregulation as evidence of increased nuclear stiffness remains indirect, as no direct bio-physical measurements of nuclear mechanics were performed. Future studies should identify direct molecular targets of allomyrinasin and establish causal relationships through pharmacological and genetic manipulation, including a comparative analysis in OSCC cell lines with contrasting *TP53* backgrounds such as YD-9 (wild-type *TP53*) or YD-8 (point mutation of *TP53* at R273H), perform flow cytometric subpopulation analysis, incorporate protein-level validation of key findings, and evaluate efficacy in orthotopic OSCC xenograft models.

## 5. Conclusions

In conclusion, allomyrinasin suppresses migration-related aggressive behavior in OSCC by inhibition of NBC activity, lamin A/C upregulation, *MMP-13* downregulation, promoting ROS accumulation, and activating pro-apoptotic signaling. These findings identify allomyrinasin as a potential anti-metastatic therapeutic candidate and provide mechanistic insights that may guide combination therapeutic strategies in OSCC.

## Figures and Tables

**Table 1:** Primer sequences used for RT-qPCR analysis.

Gene	Forward (5′→3′)	Reverse (5′→3′)
*TP* *53*	TGGGACAGCCAAGTCTGTGA	GGCCAGTTGGCAAAACATCT
*BAX*	GTGGCAGCTGACATGTTTTCTG	GCCTTGAGCACCAGTTTGCT
*BCL2*	AACTGTACGGCCCCAGCAT	GCCAAACTGAGCAGAGTCTTCAG
*MMP-2*	TGTGACGCCACGTGACAAG	GCCTCGTATACCGCATCAATCT
*MMP-7*	CGGATGGTAGCAGTCTAGGGATT	GAGGAATGTCCCATACCCAAAG
*MMP-9*	CGCTGGGCTTAGATCATTCC	GTGCCGGATGCCATTCA
*MMP-13*	TCTCGCGGGAATCCTGAA	GTCACCTCTAAGCCGAAGAAAGAC
*GAPDH*	GACCTGACCTGCCGTCTAGAAA	CCTGCTTCACCACCTTCTTGA

## Data Availability

The original contributions presented in this study are included in the article. Further inquiries can be directed to the corresponding authors.

## Abbreviations

AMP	Antimicrobial peptide
Bax	Bcl-2-associated X protein
*BAX*	BCL2-associated X apoptosis regulator (gene)
Bcl-2	B-cell lymphoma 2
*BCL2*	B-cell lymphoma 2 (gene)
BCECF-AM	2′,7′-bis-(2-carboxyethyl)-5(and-6)-carboxyfluorescein acetoxymethyl ester
BSA	Bovine serum albumin
CCD	Charge-coupled device
cDNA	Complementary DNA
COX-2	Cyclooxygenase-2
CTCF	Corrected total cell fluorescence
DAPI	4′,6-diamidino-2-phenylindole
DCFH-DA	2′,7′-dichlorofluorescin diacetate
DPBS	Dulbecco’s phosphate-buffered saline
ECM	Extracellular matrix
EDTA	Ethylenediaminetetraacetic acid
EIPA	5-(N-Ethyl-N-isopropyl) amiloride
FBS	Fetal bovine serum
FITC	Fluorescein isothiocyanate
GAPDH	Glyceraldehyde-3-phosphate dehydrogenase
iNOS	Inducible nitric oxide synthase
MMP	Matrix metalloproteinase
MTS	3-(4,5-dimethylthiazol-2-yl)-5-(3-carboxymethoxyphenyl)-2-(4-sulfophenyl)-2H-tetrazolium
NBC	Sodium bicarbonate cotransporter (Na^+^/HCO_3_^−^ cotransporter)
OSCC	Oral squamous cell carcinoma
pHi	Intracellular pH
PI	Propidium iodide
ROS	Reactive oxygen species
RPMI	Roswell Park Memorial Institute (medium)
RT-qPCR	Reverse transcription quantitative polymerase chain reaction
SD	Standard deviation
p53	Tumor protein p53 (protein)
*TP53*	Tumor protein p53 (gene)
UDG	Uracil-DNA glycosylase
